# Rapid mobilization of a virtual pediatric chronic pain clinic in Canada during the COVID-19 pandemic

**DOI:** 10.1080/24740527.2020.1771688

**Published:** 2020-08-05

**Authors:** Lisa N. D’Alessandro, Stephen C. Brown, Fiona Campbell, Danielle Ruskin, Giulia Mesaroli, Mallika Makkar, Jennifer N. Stinson

**Affiliations:** a Department of Anesthesia and Pain Medicine, Hospital for Sick Children and University of Toronto, Toronto, Ontario, Canada; b Lawrence S. Bloomberg Faculty of Nursing, University of Toronto, Toronto, Ontario, Canada; c Department of Psychology, Hospital for Sick Children, Toronto, Ontario, Canada; d Department of Psychology, York University, Toronto, Ontario, Canada; e Department of Rehabilitation Services, Hospital for Sick Children, Toronto, Ontario, Canada; f Department of Physical Therapy, University of Toronto, Toronto, Ontario, Canada; g Child Health Evaluative Sciences, Research Institute, Hospital for Sick Children, Toronto, Ontario, Canada

**Keywords:** COVID-19, pandemic, chronic pain, pediatrics, virtual care, ambulatory

## Abstract

**Background:**

Studies have been conducted describing the potential for using virtual care software during disasters and public health emergencies. However, limited data exist on ways in which the Canadian health care system utilizes virtual care during disasters or public health emergencies.

**Aims:**

Due to the need for social distancing and reduction of nonessential ambulatory services during the COVID-19 pandemic, the SickKids Chronic Pain Clinic sought to transition care delivery from in person to virtual. The virtual clinic aimed to reduce risks associated with physical contact and environmental exposure without reducing access to care itself.

**Methods:**

Harnessing of various digital tools including Ontario Telemedicine Network Guestlink, Zoom, and Microsoft Teams. The Chronic Pain Clinic Team worked together to communicate with patients and families, schedule virtual visits, establish remote access to clinical data collection tools, digitize the after-visit summary, and add resources on pain self-management to the clinic’s website.

**Results:**

The Chronic Pain Clinic successfully transitioned all clinic appointments (multidisciplinary and individual; 77 appointments) over a 2-week period to virtual care. Virtual clinics did not surpass the usual time taken pre-COVID-19, suggesting that the clinic workflow was readily adaptable to virtual care.

**Conclusions:**

Access to quality virtual care is essential to prevent chronic pain from taking a toll on the lives of patients and families. Rapid establishment of a virtual clinic without gaps in service delivery to patients is possible given institutional support and a team culture centered around collaboration and flexibility.

## RÉSUMÉ

**Contexte**: Des études ont été menées pour décrire le potentiel d’utilisation des logiciels de soins virtuels lors de catastrophes et d’urgences de santé publique. Toutefois, il existe peu de données sur les moyens par lesquels le systéme de soins de santé canadien utilise les soins virtuels lors de catastrophes ou d’urgences de santé publique.

**But**: En raison de la nécessité d’une distanciation sociale et de la réduction des services ambulatoires non essentiels pendant la pandémie de COVID-19, la clinique de douleur chronique de SickKids a cherché à assurer une transition de la prestation des soins en personne vers des soins virtuels. La clinique virtuelle visait à réduire les risques liés au contact physique et à l’exposition environnementale sans réduire l’accès aux soins en tant que tels.

**Méthodes**: Exploitation de divers outils numériques, dont le Réseau de télémédecine de l’Ontario Guestlink, Zoom et Microsoft Teams. L’équipe de la Clinique de la douleur chronique a travaillé ensemble pour communiquer avec les patients et les familles, programmer des visites virtuelles, établir un accès à distance aux outils de collecte de données cliniques, numériser le résumé après la visite et ajouter des ressources sur l’auto-prise en charge de la douleur sur le site web de la clinique.

**Résultats**: La Clinique de la douleur chronique a réussi à faire passer tous les rendez-vous de la clinique (multidisciplinaires et individuels; 77 rendez-vous) sur une période de deux semaines à des soins virtuels. Les cliniques virtuelles n’ont pas dépassé le temps habituel avant la COVID-19, ce qui indique que le flux de travail de la clinique était facilement adaptable aux soins virtuels.

**Conclusions**: L’accès à des soins virtuels de qualité est essentiel pour éviter que la douleur chronique ne fasse des ravages dans la vie des patients et des familles. L’établissement rapide déune clinique virtuelle sans interruption dans la prestation des services aux patients est possible grâce à un soutien institutionnel et à une culture d’èquipe centrèe sur la collaboration et la souplesse.

## Introduction

Chronic pain affects one in five Canadian children and youth^1^ and can negatively impact all aspects of quality of life.^2,3^ A study of pediatric chronic pain in the UK reported a mean annual cost of illness of £8000 (roughly C$14,000) per adolescent with pain and £3.8 billion (roughly C$6.7 billion) in public costs to society.^4^ Early pain management intervention can reduce pain duration, pain-related disability, and health care costs. Two surveys have revealed unacceptably high levels of undertreatment and intolerably long wait times to access interdisciplinary pain care in Canada.^5,6^ In 2013, the Ontario Ministry of Health and Long-Term Care infused C$3.5 million to improve access to pediatric chronic pain care in Ontario, the most populous province in Canada.^7^

The optimal treatment model for chronic pain in children is a multidisciplinary “3 P” approach that involves pharmacological, physical, and psychological therapies.^8^ The Chronic Pain Program at the Hospital for Sick Children (SickKids) in Toronto is the largest pediatric pain program in Canada. Between 2018 and 2019, 2101 appointments were completed, including 210 new referrals. A multidisciplinary team comprised of an anesthesiologist, advanced practice nurse, psychologist, and physiotherapist together see all new patients (90-min appointments) and follow-up patients (45-min appointments). In addition, the team has a dedicated psychiatrist, occupational therapist, rehabilitation therapy assistant, patient care information coordinator, and data analyst. A new patient appointment includes assessing pain characteristics, general health, and social history; a brief mental health consult; and a physical examination by the anesthesiologist and physical therapist. After the physical examination, the team regroups to determine the diagnosis and generate treatment recommendations using the “3 P” approach; this information is finally shared with the patient and his or her family. Subsequent treatments are typically provided in person at SickKids. Alternatively, treatments may be delivered virtually through Telemedicine or Guestlink^TM^ (via Ontario Telemedicine Network, OTN) or in person through health care providers (HCPs) in the patient’s community.

Previous work has specifically described the potential for using virtual care software during disasters and public health emergencies,^9^ but such systems are difficult to develop overnight. The OTN already supports two secure videoconferencing software programs: telemedicine, which requires both patients and HCPs to be in health care facilities in order to connect, and GuestLink, through which patient and HCP can connect from anywhere, including their homes. Historically, the SickKids Chronic Pain Clinic has utilized telemedicine and GuestLink for patient care videoconferencing and Zoom for knowledge mobilization and outreach. Zoom software is also used to deliver Project ECHO Ontario^TM^ (http://www.echoontario.ca/).

In 2019, the hospital developed a virtual care steering committee with a mandate to provide guidance and oversight in advancing the implementation and scaling of virtual care across SickKids. Moreover, the hospital’s recently announced 2020–2025 strategic plan includes a mandate to transcend geography through virtual care, to allow for a seamless child and family experience. Though our Pediatric Chronic Pain Clinic already had extensive knowledge of and practice delivering virtual care, the COVID-19 pandemic not only provided an urgent opportunity but also necessitated moving ambulatory in-person visits to virtual care within an incredibly short time frame. This article describes the context and process of rapidly mobilizing a virtual Pediatric Chronic Pain Clinic during the COVID-19 pandemic.

## Context

In early January 2020, the SickKids Executive Leadership Team established a working group to address rising concerns surrounding the growing COVID-19 pandemic. Two cases of COVID-19 were identified in Ontario a few weeks later, prompting the implementation of a patient and family high-risk screening alert. This alert was the impetus to appoint the Chronic Pain Clinic’s advanced practice nurse as the COVID-19 team champion. As the number of confirmed COVID-19 cases grew in the province, a hospital-wide approach was initiated, resulting in cancellations of all nonurgent in-patient and ambulatory visits and all elective surgeries. Within 24 h of this decision, the SickKids Chronic Pain Team champion led the creation of a plan to transition nearly all new and follow-up appointments and individual treatment sessions (psychology, physical therapy, occupational therapy, psychiatry, and medication check-ins) from in-person to virtual care. This plan was implemented, evaluated, and modified over the course of 2 weeks.

## Steps and Resources

### Priority Setting in Care

We converted our routine weekly in-person case rounds to urgent COVID-19 response virtual meetings using Zoom. Agenda items included review of COVID-19 organizational response, establishment of group trust in rapid decision making, logistics of switching to virtual appointments, and peer support. The team consensus was to move all in-person visits, including new and follow-up appointments and treatment sessions, to virtual care. Patient appointments booked over the subsequent 2 weeks were triaged for virtual care appropriateness. Only urgent chronic pain patients for whom virtual care would not be appropriate—typically limited to patients with pain related to complex regional pain syndrome or cancer or patients in palliative care—would be offered in-person appointments.

### Needs Assessment

Needs assessments were conducted to shine light on the current state of virtual care across the hospital and staff members’ proficiency with using software for virtual care. The need for professional flexibility was heightened, because triaging urgent assessments and treatments, scheduling virtual appointments and meetings using videoconferencing software, and timely patient and family communication demanded a team-wide effort. Moreover, staff required refreshers on using the software and on preexisting organizational policies and procedures related to software use for clinical purposes. Table 1 outlines the software utilized at SickKids.

**Table 1.** Virtual care platforms and their clinical utility

Virtual platform	Utility
Ontario Telemedicine Network GuestLink	The telemedicine program at SickKids words in collaboration with the Ontario Telemedicine Network as a hospital-approved virtual care modality for conducting virtual patient appointments The GuestLink tool is used to conduct videoconferencing for patient appointments in their desired location
Telemedicine	Virtual patient appointments
Microsoft Teams	Team chat and videoconferencing software used for internal communication (patient case conferences pre, during, and post virtual patient appointment)
Zoom	Videoconferencing software used for internal communication: clinical operations and workflow and team meetings to review clinical care
WhatsApp	Team chat used for internal communication: COVID-19 updates, technology troubleshooting, other business

### Privacy and Confidentiality Considerations

Time constraints dictated the utilization of virtual care platforms to ensure no disruption in patient care. For instance, though we had the option to use Zoom for Healthcare, we chose Guestlink because we already had extensive experience with using it in the past to deliver care. Furthermore, Guestlink is designed to operate in accordance with the requirements of the Personal Health Information Protection Act (PHIPA), S.O. 2004, and Regulation 329/04. We sent emails to patients outlining the provisions taken by our clinic staff to ensure privacy and confidentiality during videoconference appointments to alleviate any potential concerns. For example, each appointment is assigned a secure, encrypted, password-protected videoconference link. Additionally, the host can lock the videoconference once it has commenced to prevent unwanted or unexpected individuals from joining the session. Both patients and staff also availed themselves of support and resources surrounding privacy on the OTN website (https://support.otn.ca/en/members/privacy-toolkit).

### Clinical Activity

Patients already booked to visit the Chronic Pain Clinic in person were rebooked to virtual care by the clinic’s patient care information coordinator. Patients were contacted personally to explain the need to reschedule to virtual care and to address questions surrounding Guestlink by both telephone and by email. Each clinician’s duties remained the same as pre-COVID-19, with the exception of one physical therapist, who was reassigned part-time to in-patient units. The clinic remained otherwise fully staffed during the transition to virtual care.

The clinic used the same model of patient assessment following the transition to virtual care. Each virtual appointment remained multidisciplinary: anesthesiologist, advanced practice nurse, psychologist, physical therapist, and psychiatrist were all present, for a total of six to seven people in each appointment, including the patient and his or her family. Length of new patient appointments and follow-up appointments remained 90 min and 45 min. Clinics did not surpass the usual time taken pre-COVID-19, suggesting that our clinic workflow was readily adaptable to virtual care.

Clinic staff used Microsoft Office Teams (MST) to review patient charts prior to commencing the Guestlink videoconference with each patient. As mentioned above, the appointment typically consists of an appraisal of pain characteristics, general health, social history, and mental health and a physical examination by the anesthesiologist and physiotherapist. A few modifications were made such that these goals could still be achieved in a virtual context. For instance, during the physical exam, only the anesthesiologist and physiotherapist remained with the patient in Guestlink; the other team members signed off OTN to meet in MST. Additionally, some components of the typical physical exam, such as a neurological exam including deep tendon reflexes, were not carried out.

Other components were modified as necessary. For example, palpation and sensation of structures surrounding the painful area by light touch and deep palpation, which is normally carried out by a clinician, was instead carried out by the patient or parent. The virtual physical examination also included observation of the painful area, active movements of the surrounding joints and limbs, functional movements (i.e., squatting), and balance (e.g., ability to stand on one leg). These tasks were first demonstrated by the physiotherapist and then carried out by the patient. Height and weight were reported by the patient or parent. After the physical examination, the entire team regrouped in MST to discuss findings while the patient remained in the Guestlink appointment. After the team discussed the findings and drafted recommendations, they regrouped in the Guestlink appointment to share recommendations and next steps in care with the patient and caregiver.

### Patient Data Collection

Our clinic relies on a clinical Pediatric Pain Registry comprised of validated patient-reported outcome surveys on anxiety, depression, physical functioning, and other important domains of health and quality of life. Administered online via REDCap, the registry allows clinicians to better understand the impact of pain on patients’ lives prior to their appointment. Prior to workflow changes due to COVID-19, new patients completed registry surveys on clinic-provided iPads in the waiting room before their appointment. Follow-up patients received the surveys via email 2 weeks before their appointment. As part of the clinic’s adaptation to COVID-19, all new and follow-up patients have received links to their surveys via email before their appointments, allowing this information to be available during their virtual appointments.

### Patient Education

Historically, at the end of in-person appointments, the individualized treatment recommendations are incorporated into an after-visit summary (AVS), which also contains resources to help with pain education and pain self-management and contact information for members of the patient’s circle of care. The AVS is usually printed directly from our electronic health record (EPIC) and given to patients and caregivers for reference. However, with the transition to virtual appointments, the AVS was adapted for email distribution to families. This involved changing clinicians’ contact information from phone numbers to email addresses, adding mental health crisis resources, and adding chronic pain virtual self-management tools. Lastly, in anticipation of potential reductions in staff and delivery of individualized treatments, our team vetted and uploaded a significant number of chronic pain self-management resources for patients to the clinic’s website (http://www.sickkids.ca/Anesthesia/programsservices/Chronic-Pain-Clinic/index.html).

## Conclusions

This article describes the successful mobilization of a virtual Pediatric Chronic Pain Clinic during the COVID-19 pandemic. Failure to rapidly adopt virtual care practices would have been greatly detrimental to the health-related quality of life of children and adolescents with chronic pain and their families. Virtual care maintained access to care for children and their families despite the pandemic and eliminated the need to travel to the hospital, thereby reducing both risk of exposure to or transmission of the novel coronavirus as well as the cost of travel itself.

Sadly, despite adoption of virtual care protocols, the pandemic will still impact the health and well-being of young people suffering from chronic pain due to closure of schools, libraries, and other nonessential services, which are essential to improving physical functioning and reducing the impact of chronic pain. In addition, there remain challenges to virtual clinics, including unstable wireless networks, lack of access to required software and hardware for both patients and staff working remotely, and access to virtual clinics, given that a digital divide still exists in Canadian communities.^10^ Conducting physical examinations remotely is also challenging, especially in patients with neuropathic pain. We have also temporarily suspended our patient screener for suicidality while our mental health colleagues form protocols for responding to severe distress disclosed during virtual care visits. In light of these challenges, it is apparent that new, tailored approaches toward virtual care are greatly needed.

Nevertheless, the move to virtual care highlighted our clinic’s ability to transform care and service through creative and proactive thinking. Table 2 summarizes the number of appointments within a 2-week time frame that were transitioned to virtual care.

**Table 2.** Type and numbers of virtual care appointments

Type of appointment	Virtual care	Face-to-face care
Interdisciplinary new	4	2 (1 patient with language barrier, 1 with cancer pain)
Interdisciplinary follow up	13	0
Rapid Access CRPS Clinic^a^	2	0
Rehabilitation services (physical therapy, occupational therapy, rehab assistant)	35	0
Psychology/psychiatry/advanced practice nurse	33	0

^a^ An interdisciplinary clinic aimed at assessing patients who are suspected to have CRPS within one week of receiving referral.

CRPS = complex regional pain syndrome.

### Staff and Patient Experience

Staff were given the opportunity to share their personal and patient experiences with virtual care each week. Initial staff comments included concerns around “the potential for discrimination to those patients without access to technology required for virtual care.” One staff member voiced that they “miss the nonverbal cues [they] receive in psychology in-person clinic appointments,” and another noted that it is “hard to see in the physical exam if there is swelling or color changes.” Some staff expressed that they miss seeing their teammates in person as well.

Technical challenges arose regularly, including lack of bandwidth needed for Guestlink, causing a decrease in both audio and video quality for both staff and patients. It was also difficult to create a safe space for patients to talk about sensitive topics such as substance use or other concerns that they wanted to keep private. Clinic staff also reported “virtual care fatigue,” characterized by feelings of fatigue after sitting in front of a computer screen for the duration of clinic. Although staff continue to share their virtual care experiences during weekly team meetings, a more formal staff satisfaction survey has been created to better understand existing issues and to assess the feasibility, implementation, and sustainability of conducting clinics virtually. We hope to share the results of this survey in future work.

Historically, patients were also given the opportunity to share their feedback and experience by completing a satisfaction survey before leaving clinic. The clinic is determined to assess patient satisfaction related to equipment and technical issues, communication and rapport, clinical assessment, and program evaluation. Therefore, we updated our patient satisfaction survey to include virtual care considerations within a week of migrating to virtual care.

Preliminary feedback from families has mostly been positive. One patient expressed immense gratitude for the opportunity to receive care virtually and said, “I was scared it might be canceled and wouldn’t get help.” On the other hand, one patient preferred in-person appointments and declined psychology appointments until they could be offered again in person. This speaks to the existing variety in patient preference as it pertains to methods of care delivery.

### Key Enablers

A number of key enablers allowed the team to respond within such a short time frame. These include our COVID-19 team champion, our existing virtual care infrastructure, our robust and dedicated 24/7 IT service, and processes made possible by the SickKids Executive Leadership Team’s 2019 mandate and the SickKids 2020–2025 strategic plan. For those HCPs challenged with meeting the needs of their patients in pain, we suggest reviewing the pertinent provincial telehealth program to understand its feasibility for both patient and HCP needs. OTN alleviated the need to create educational material around security and privacy for patients and staff, which in turn decreased administrative work.

Our multidisciplinary team continues to champion innovation and flexibility; our highly cohesive working style ensured the success of this transition. Constant emphasis on the clinic’s goal to support and empower children and families in the self-management of chronic pain motivated the team to rapidly facilitate almost 100% virtual care. Our team worked around the clock to realize the transition, with no gaps in service experienced by our patients. Lastly, SickKids Executive leadership’s quick response to COVID-19, transparency in communication, and faith in staff members to actualize creative solutions provided the institutional support required to carry out our team’s plan.

### Future Implications

Our rapid transition has underscored the importance of providing freely available resources to help patients learn pain self-management skills. Innovations in virtual self-management and care delivery models can also help reduce barriers to accessing care, including long-distance travel to hospital and time away from work and school. We also need to improve the knowledge and comfort level of primary care providers to care for these patients in their local communities through innovative programs such as Pediatric Project ECHO, which provides virtual mentorship to community HCPs caring for complex pediatric pain patients.^11^

This pandemic will likely change the way we care for pediatric chronic pain patients in the future. We need to seize this opportunity to harness the full potential of virtual care without compromising access to and quality of care. As a next step, we are evaluating the impact of the move to virtual care on patients and their families by conducting an evaluation of the strategies documented in this report.
